# The effect of aging on glucose metabolism improvement after Roux-en-Y gastric bypass in type 2 diabetes rats

**DOI:** 10.1038/s41387-022-00229-9

**Published:** 2022-12-23

**Authors:** Weijie Chen, Haixin Yin, Jianchun Xiao, Wei Liu, Qiang Qu, Fengying Gong, Xiaodong He

**Affiliations:** 1 Department of General Surgery, Peking Union Medical College Hospital, Chinese Academy of Medical Sciences, Shuaifuyuan 1#, Beijing, 100730 PR China; 2 Department of Endocrinology, Key Laboratory of Endocrinology of the Ministry of Health, Peking Union Medical College Hospital, Chinese Academy of Medical Sciences, Shuaifuyuan 1#, Beijing, 100730 PR China

**Keywords:** Type 2 diabetes, Obesity

## Abstract

**Background:**

This study aimed to investigate the effect of aging on glucose metabolism improvement after Roux-en-Y gastric bypass (RYGB) in rat models with type 2 diabetes mellitus (T2DM).

**Methods:**

Twenty aged Goto-Kakizaki rats were randomly assigned into RYGB-A group and sham RYGB (SR-A) group, and 10 adult Goto-Kakizaki rats also accept RYGB procedures (RYGB-Y). Glucose metabolism, resting energy expenditure (REE), glucagon-like peptide-1 (GLP-1) and total bile acid level were measured.

**Results:**

RYGB could significantly improve glucose metabolism in aged diabetic rats. The fasting blood glucose level in the RYGB-A group decreased from 15.8 ± 1.1 mmol/l before surgery to 12.3 ± 1.5 mmol/l 16 weeks after surgery (*P* < 0.01), and the AUC_OGTT_ value decreased from 2603.9 ± 155.4 (mmol/l) min to 2299.9 ± 252.8 (mmol/l) min (*P* = 0.08). The decrease range of fasting blood glucose in the RYGB-A group was less than that in the RYGB-Y group (20.5% ± 6.5% vs. 40.6% ± 10.6%, *P* < 0.01), so is the decrease range of AUC_OGTT_ value (11.6% ± 14.8% vs. 38.5% ± 8.3%, *P* < 0.01). Moreover, at the 16th postoperative week, the increase range of REE of the RYGB-A group was lower than that of the RYGB-Y group (15.3% ± 11.1% vs. 29.1% ± 12.1%, *P* = 0.04). The increased range of bile acid of the RYGB-A group was less than that of the RYGB-Y group (80.2 ± 59.3 % vs.212.3 ± 139.0 %, *P* < 0.01). The GLP-1 level of the RYGB-A group was less than that of the RYGB-Y group (12.8 ± 3.9 pmol/L vs. 18.7 ± 5.6 pmol/L, *P* = 0.02). There was no significant difference between the RYGB-A group and the RYGB-Y group in the level of the triiodothyronine level.

**Conclusions:**

RYGB could induce a glucose metabolism improvement in aged diabetic rats, and aging might moderate the effect of RYGB.

## Introduction

Type 2 diabetes mellitus (T2DM) is a worldwide chronic metabolic disease with potentially severe complications and socioeconomic effects [1]. It is considered as an age-related pathology, because aging leading to insulin resistance, dyslipidemia and obesity, which are high risk factors for T2DM [2]. In people over the age of 65 years, more than one-quarter of older adults have T2DM and about one-half have prediabetes [3]. And the number of aged adults living with T2DM and prediabetes is increasing rapidly, it was expected to increase by 4.5-fold between 2005 and 2050 [4]. However, aged adults are also at high risk of hypoglycemia for many reasons, that includes progressive renal insufficiency and insulin deficiency necessitating insulin therapy [5]. Therefore, aged adults with T2DM have a different treatment targets and therapeutic approaches [3].

Roux-en-Y gastric bypass (RYGB) is a kind of recommended bariatric surgery, which has been proposed as an appropriate therapy for T2DM resolution [6]. The gastrointestinal reconstruction reduces food intake in the early stage and leads to rapid delivery of chyme to the distal intestine. Satiety hormones, like glucagon-likepeptide-1 (GLP-1) and bile acids changes significantly after RYGB [7, 8]. They could lead to decrease energy intake, improve insulin resistance, increase sympathetic nerve stimulation of peripheral tissues and resting energy expenditure (REE) [9].

Aged organisms would lose their ability to modulate the adaptive homeostatic response, which is transient or continuous adjustments for internal and external conditions [10]. And the compensatory basal increase of stress-responsive enzymes would further compress the maximal range of responses, consequently might diminish the effect of hypoglycemic treatment [10]. Patients seeking for metabolic surgery are usually younger, they have a shorter duration of diabetes and lower proportion of insulin dependency [11]. There were few studies about glucose metabolism changes in aged adults with T2DM after RYGB. And the defined effect of aging has not been clear. Therefore, we aimed to measure the glucose metabolism changes in aged T2DM rat models after RYGB.

## Materials and methods

### Animals

Twenty 12-months-old and ten 2-months-old male Goto-Kakizaki (GK) rats, represent aged and young T2DM patient respectively, were obtained from National Rodent Laboratory Animal Resources (Shanghai, P. R. China) [12]. Because of the accumulation of multiple variations, GK rat models could simulate the pathogenesis of T2DM in human, and be widely used for non-obesity T2DM investigation [13]. They were fed with a 5% fat rat chow diet and tap water ad libitum before operation, and housed individually in a rat cage at a temperature of 22 °C with a 12 h light-dark cycle.

After 1-week of acclimation, twenty 12-months-old rats were randomly divided into 2 groups, each group with 10 rats: the RYGB-A group and the sham RYGB (SR-A) group. And 2-months-old rats were assigned to the young control group (RYGB-Y).

### Surgical procedures

**Fig. 1:**
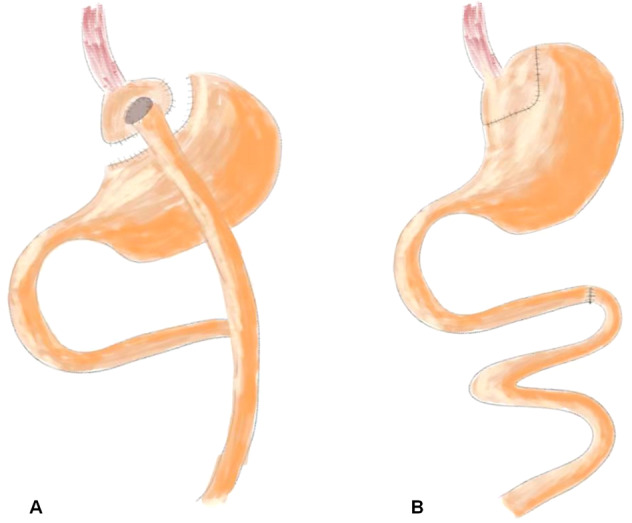
The schema of surgical procedure. **A** The schema of Roux-en-Y gastric bypass. The food bypass the most stomach and reach to mid-jejunal. **B** The schema of sham Roux-en-Y gastric bypass. The sham surgery involved same incisions or transections without food bypass.

RYGB was performed in the RYGB-A group and the RYGB-Y group under anaesthetization using 10% chloral hydrate solution. The rat stomach was transected to generate a small pouch (about 5–10% by volume), and anastomosed to the mid-jejunal. The most distal stomach, duodenum and proximal jejunum were passed by and isolated from the digestive flow as publication previously (Fig. 1) [14]. As a result, distal jejunal received an expedited delivery of mostly undigested nutrients, while duodenum and proximal jejunum were. The SR-A group rats received a sham surgery, which involved same incisions or transections of RYGB and in situ anastomosis (Fig. 1). At 16th week after operation, all rats were sacrificed using CO2 followed by decapitation. All surgical procedures were approved by the ethics committee of our hospital (XHDW-2019-029). All applicable national guidelines for the use and care of animals were followed.

### Food intake and bodyweight

After fed with nonresidue diet (Chicago, USA) for 2 days and an overnight (at least for 10 h) fasting, RYGB was performed on the RYGB-A group and SR-A group rats. Two hours after surgery, the rats had free access to water and non-residue diet afterwards. After 1 week, they were fed 5% fat rat chow diet. Three group rats were matched according to fasting glucose, and paired fed with a same amount of food in 24 h. The maximum food intake was measured after overnight fasting, rats were fed without food restriction in 24 h.

### Oral glucose tolerance test

Oral glucose tolerance tests (OGTTs) were used for insulin resistance investigation, and performed postoperatively at the 2nd, 12th, and 16th weeks [15]. After an overnight fasting, each rat took an oral administration of glucose solution (2 g glucose per 1 kg body weight), then the blood glucose of each rat was measured at the 15th, 30th, 60th, and 120th minute using a glucometer (Roche One Touch^®^ Ultra, Lifescan, Johnson & Johnson, Milpitas, USA). The area under the curve for the OGTT (AUC_OGTT_) was calculated by a trapezoidal integration method.

### Basal metabolic rate

The REE of each rats was measured by an indirect calorimetry method [16]. After an overnight fasting, each rat was housed in a transparent metabolic chamber. The metabolic chamber contained soda lime (Shanghai, P. R. China) to absorb carbon dioxide exhaled by rats, and was added oxygen to equilibrate the pressure within the system. During the day between 8.00 and 11.00 h, the volume added into the metabolic chamber was measured under a quiet condition. And the REE was calculated by dividing the volume of oxygen consumed by the duration and the body weight of the rat [17].

### Biochemical tests

At postoperative 2nd, 12th, and 16th week, blood samples were collected from rat tail veins and stored in −20 °C. The level of plasma bile acids (Minneapolis. USA), triiodothyronine (Wuhan, P. R. China) and GLP-1 (Shanghai, P. R. China) were measured using enzyme-linked immunosorbent assays.

### Statistical analysis

Continuous variables are expressed as the means ± standard deviations. One-way ANOVA and the Tukey-Kramer test (SPSS 19, SPSS, Inc, Chicago, USA) were used to compare between three groups, and the paired *t* test was used to compare the state before surgery and after surgery. The probability value less than 0.05 was considered as statistically significant.

## Results

### Animal model

All surgical procedures were performed successfully in three group rats. In the RYGB-A group, seven rats survived at the 16th postoperative week. A rat died from intraperitoneal infection at the 2nd postoperative week, two rats died from intestinal obstruction at the 6th postoperative week and 12th postoperative week respectively. In the SR-A group, eight rats survived, two rats died from intestinal obstruction at the 4th and 6th week postoperatively. And in the RYGB-Y group, eight rats survived, two rats also suffered from intestinal obstruction and died at 4th and 12th week. There were no other severe complications observed in three groups.

### Food intake and body weight

**Fig. 2:**
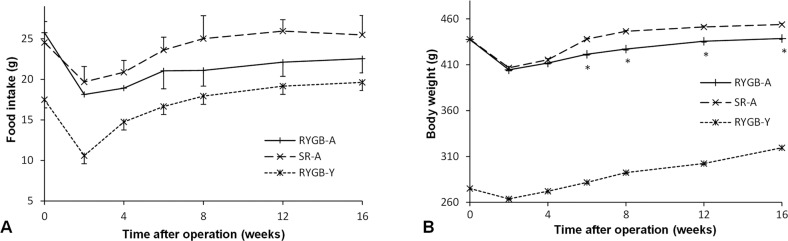
The food intake and body weight curve of three groups. **A** The food intake curve of the RYGB-A group, the SR-A group and the RYGB-Y group. There was no significant difference in the food intake between the RYGB-A group and the SR-A group after surgery. **B** The body weight curve of the RYGB-A group, the SR-A group and the RYGB-Y group. The body weight of the RYGB-A group was significantly less than the SR-A group. The asterisk means significantly different compared to the SR-A group (*P* < 0.05). RYGB-A, aged rat with Roux-en-Y gastric bypass. SR-A, aged rat with sham Roux-en-Y gastric bypass. RYGB-Y, young rat with Roux-en-Y gastric bypass.

Before surgery, there was no significant difference in food intake between RYGB-A group and SR-A group (25.8 ± 2.6 g vs. 24.5 ± 1.8 g, *P* = 0.24). The food intake of the RYGB-A group was more than the RYGB-Y group (25.8 ± 2.6 g vs. 17.5 ± 2.8 g, *P* < 0.01) as the different body weight. After surgery, three group rats were paired fed to ensure the similar amount of food intake. If the food supply was not restricted, there was no significant difference in the maximum 24-hour food intake between three groups at the 16th postoperative week (*P* = 0.06, Fig. 2).

With regard to the body weight, there was no significant difference between RYGB-A group and SR-A group before surgery (437.5 ± 13.2 g vs. 437.8 ± 12.2 g, *P* = 0.96). Because of different ages, the body weight of the RYGB-A group was more than that of the RYGB-Y group (437.5 ± 13.2 g vs. 275.2 ± 6.5 g, *P* < 0.01). After surgery, the body weight of the RYGB-A group was less than that of the SR-A group (438.6 ± 12.6 g vs. 453.9 ± 8.4 g, *P* = 0.02), but still more than that of the RYGB-Y group at the 16th postoperative week (438.6 ± 12.6 g vs. 319.5 ± 13.8 g, *P* < 0.01, Fig. 2).

### Glucose improvement

**Fig. 3:**
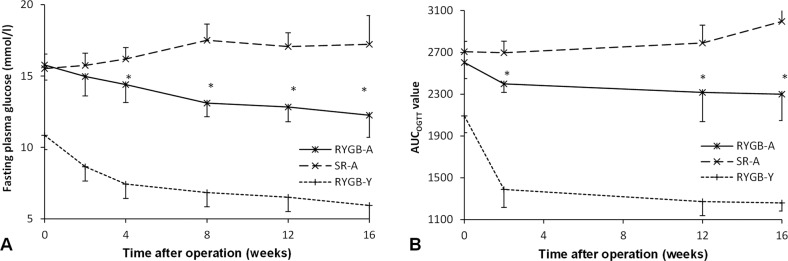
The effect of RYGB on the improvement of blood glucose. **A** The fasting glucose curve of three groups. RYGB-A rats showed a lower fasting glucose level compared to the SR-A group after surgery. **B** The AUC_OGTT_ during the postoperative period. Rats of the RYGB-A group showed amelioration of diabetes after surgery, and their AUC_OGTT_ value was less than that of the SR-A group. The asterisk means significantly different compared to the SR-A group (*P* < 0.05). AUC, areas under curve. RYGB-A, aged rat with Roux-en-Y gastric bypass. SR-A, aged rat with sham Roux-en-Y gastric bypass. RYGB-Y, young rat with Roux-en-Y gastric bypass. OGTT, oral glucose tolerance test.

Before surgery, there was no significant difference in the fasting blood glucose and AUC_OGTT_ value between RYGB-A group and the RYGB-Y group. After surgery, the fasting blood glucose level in the RYGB-A group decreased from 15.8 ± 1.1 mmol/l to 12.3 ± 1.5 mmol/l at 16th postoperative week (*P* < 0.01), and the AUC_OGTT_ value decreased from 2603.9 ± 155.4 (mmol/l) min to 2299.9 ± 252.8 (mmol/l) min (*P* = 0.08). While the fasting blood glucose in the SR-A group increased from 15.5 ± 1.0 mmol/l to 17.2 ± 2.0 mmol/l (*P* = 0.04), and the AUC_OGTT_ value increased from 2706.5 ± 98.4 (mmol/l) min to 2996.0 ± 152.0 (mmol/l) min (*P* < 0.01). The curves of the fasting blood glucose and AUC_OGTT_ are shown in Fig. 3.

The T2DM amelioration was more significant in the RYGB-Y group. The fasting blood glucose decreased by 40.6% ± 10.6% in the RYGB-Y group, while by 20.5% ± 6.5% in the RYGB-A group (*P* < 0.01). The AUC_OGTT_ value decreased by 38.5% ± 8.3% in the RYGB-Y group, while by 11.6% ± 14.8% in the RYGB-A group (*P* < 0.01).

### Basal metabolic rate

**Fig. 4:**
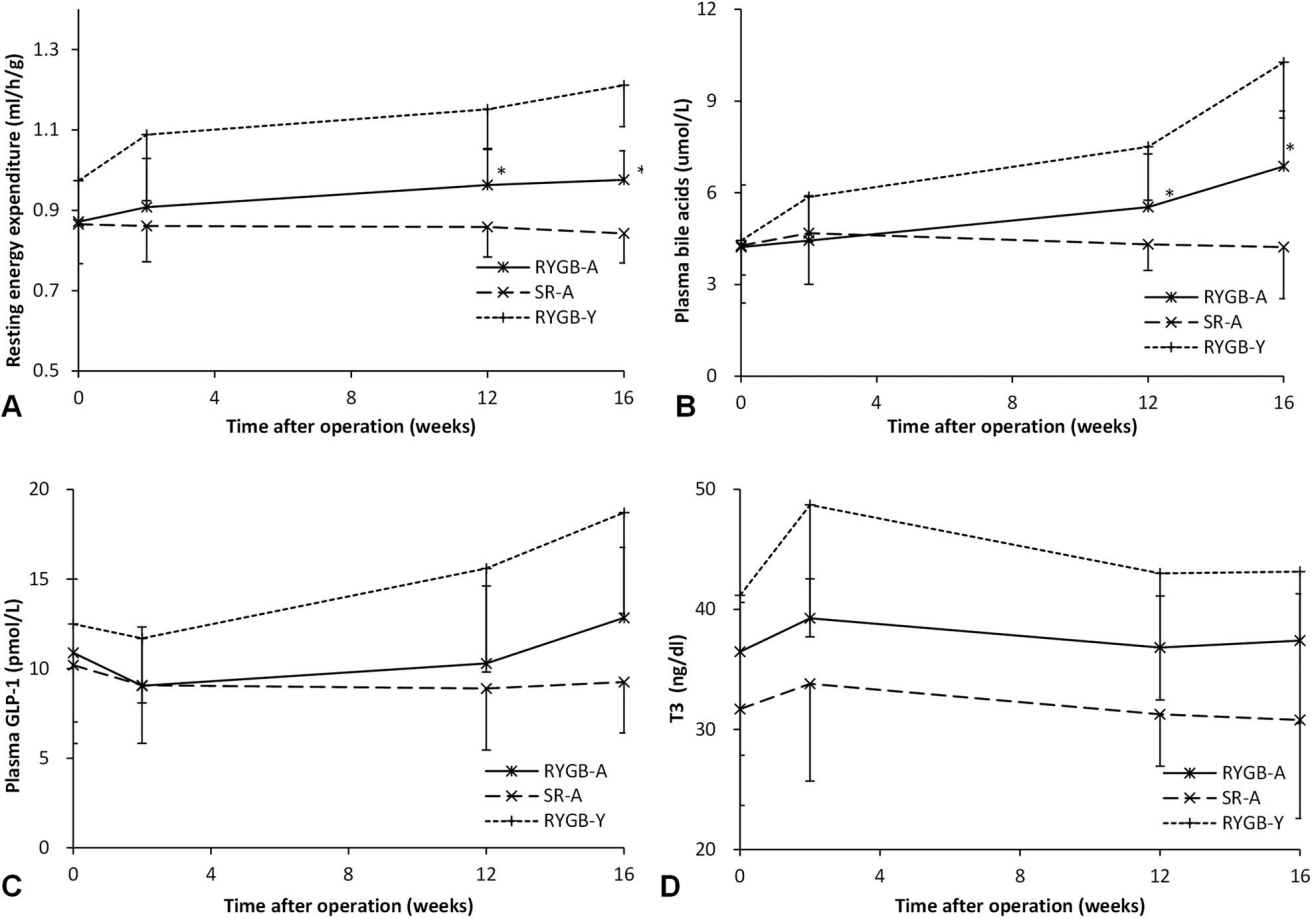
Changes of resting energy expenditure, plasma bile acid level, GLP-1, and triiodothyronine. **A** The curve of resting energy expenditure. The resting energy expenditure of the RYGB-A group was significantly more than that of the SR-A group after surgery. **B** The plasma bile acid level of three groups. The bile acid level of the RYGB-A group was elevated significantly after surgery. **C** The plasma GLP-1 level of three groups. The GLP-1 level of the RYGB-A group at the 16th postoperative week was similar with that of the SR-A group. **D** The plasma triiodothyronine level of three groups. The level of triiodothyronine in the RYGB-A group was similar with that of the SR-A group. The asterisk means significantly different compared to the SR-A group (*P* < 0.05). RYGB-A, aged rat with Roux-en-Y gastric bypass. SR-A, aged rat with sham Roux-en-Y gastric bypass. RYGB-Y, young rat with Roux-en-Y gastric bypass. T3, triiodothyronine.

Before surgery, there was no significant difference between the RYGB-A group and the SR-A group (*P* = 0.89). After surgery, the REE of the RYGB-A group increased from 0.87 ± 0.11 ml/h/g before surgery to 0.98 ± 0.07 ml/h/g at the 16th postoperative week (*P* < 0.01, Fig. 4). While the REE of the SR-A group decreased from 0.87 ± 0.10 ml/h/g before surgery to 0.84 ± 0.11 ml/h/g (*P* = 0.13). The REE of the RYGB-A group at the 16th postoperative week was significantly more than that of the SR-A group (0.98 ± 0.07 ml/h/g vs. 0.84 ± 0.11 ml/h/g, *P* < 0.01).

The REE of the RYGB-Y group also increased after surgery (1.21 ± 0.10 ml/h/g vs. 0.97 ± 0.11 ml/h/g, *P* < 0.01). The increase range of REE of the RYGB-A group was 15.3% ± 11.1%, that is lower than the increase range of REE of the RYGB-Y group (29.1% ± 12.1%, *P* = 0.04).

### Biochemical tests

There was no significant difference in the bile acid level between three groups before surgery (*P* = 0.95). After surgery, the bile acid level in the RYGB-A group increased from 4.2 ± 1.6 µmol/L before surgery to 6.9 ± 1.3 µmol/L at the 16th postoperative week (*P* < 0.01, Fig. 4), while the bile acid level in the SR-A group did not change significantly (*P* = 0.89), it was less than that of the RYGB-A group (*P* < 0.01). Although the bile acid in the RYGB-Y group also increased from 4.4 ± 2.0 µmol/L to 10.3 ± 1.8 µmol/L, the increase range of the RYGB-A group was less than that of the RYGB-Y group (80.2 ± 59.3 % vs.212.3 ± 139.0 %, *P* < 0.01).

There was no significant difference in the GLP-1 level between three groups before surgery (*P* = 0.54). After RYGB, the GLP-1 level of the RYGB-A group did not change significantly (*P* = 0.11), it was 10.9 ± 4.1 pmol/L before surgery and 12.8 ± 3.9 pmol/L at the 16th postoperative week. And the GLP-1 level of the RYGB-A group at the 16th postoperative week was similar with that of the SR-A group (*P* = 0.12), but less than that of the RYGB-Y group (12.8 ± 3.9 pmol/L vs. 18.7 ± 5.6 pmol/L, *P* = 0.02).

There was no difference in the level of triiodothyronine between three groups before surgery (*P* = 0.20). After surgery, the level of triiodothyronine in the RYGB-A group did not change significantly (36.5 ± 12.4 ng/dl vs. 37.4 ± 10.3 ng/dl*, P* = 0.96). The level of triiodothyronine in the RYGB-A group was similar with that of the SR-A group (*P* = 0.24), and the RYGB-Y group (*P* = 0.31) at the 16th postoperative week.

## Discussion

Adaptive homeostasis is a highly conserved physiological process occurred in cells, tissues, organisms and the whole body. Even an instantaneous mild internal or external processes could generate cellular perturbations, activate various signaling pathways and result in transient changes of gene expression and stress resistance [18]. These dynamic responses would lead to continual homeostatic adjustments of whole body to adapt the internal or external environment changes, which include caloric restriction/fasting, exercise-induced stress, hypoxia, oxidative stress, osmotic stress, mechanical stress, and behavioral stress [19].

RYGB changes normal gastrointestinal structure, brings bypass of chyme and leads to hormone changes and internal perturbations for glucose metabolism. The fasting blood glucose and the AUC_OGTT_ value decreased significantly in the RYGB-Y group. Meanwhile, the bile acids and the GLP-1 level increased. This was consistent with the previous literature [20, 21]. The total bile acids level is elevated, that might be the cause of glucose metabolism improvement. The reabsorption of bile acids in the distal ileum is changed by gastrointestinal reconstruction [22]. The early arrive of bile could expedite the enterohepatic cycling of bile acids. And circulating bile acids activate G protein-coupled receptor TGR5 and farnesoid X receptor in different organs [23, 24], directly affect on mitochondrial uncoupling, increase glucose intake and activate brown adipose tissue and muscle [25]. Consequently, energy expenditure is increased and the glucose metabolism is improved [26]. The level of triiodothyronine did not change significantly, that indicated the role of bile acid on the energy expenditure. The increased REE might result from the higher level of circulating bile acid level. Moreover, enteroendocrine L-cells could be activated by stimulation of TGR5, and increase GLP-1 secretion. GLP-1 could improve liver and pancreatic function and also enhance glucose tolerance [24].

There are a lot of evidences that adaptative homeostasis declines with age. As a matter of fact, aging is associated with a twofold detrimental impact on adaptive homeostasis [19]. It impairs the ability to activate or modulate various adaptive responses. Normally, cellular perturbations caused by both physiological and pathological processes generate the endoplasmic reticulum stress response, that is also called the unfolded protein response [27]. During the period of cellular duress, chaperone proteins are essential for endoplasmic reticulum, and involve in proper protein folding and cellular proteome maintainment. However, aging declines the activation of adaptive arm of the unfolded protein response resulting from age-related structural changes of endoplasmic reticulum and the gradual loss of chaperone proteins. For instance, glucose regulated protein 78 (GRP78), serving as chaperone proteins, was found a significant reduction in expression in hepatic tissue from 22-month old mice [28]. And multiple tissues from aged rat showed significant decrease expression in GRP78, including hippocampus, cerebellum, cortex, liver, lung, heart, kidney and spleen [29, 30]. Besides, GRP94 and other chaperone proteins were also found reduced in these tissues [31].

Expression changes of chaperone proteins modulate insulin sensitivity and glucose homeostasis. The decline of GRP chaperone proteins level is related with metabolic disorders, and upregulation of GRP chaperone proteins improve insulin resistance in the diabetic prone mice [32, 33]. Administration of endoplasmic reticulum chaperones could decrease weight gain and restore insulin sensitivity in diabetic mice [34]. GRP78 heterozygous mice showed lower prevalence of insulin resistance and increased glucose homeostasis compared to wild-type control [35]. Moreover, decreased GRP78 expression in adipose tissue was found in patients who underwent gastric bypass surgery [36]. In our study, although improvement of glucose metabolism was also found in the aged diabetic rats, the bile acid increased, the GLP-1 was enhanced to secrete, and REE increased after RYGB, the improvement range of glucose metabolism of the RYGB-A group was less than that of the RYGB-Y group, and the increase range of REE, bile acids and GLP-1 was also less than that of the RYGB-Y group. These results indicate the detrimental effect of aging and declined adaptative homeostasis.

The main limitation of our study is that observational researches in diabetic rat models lack causality investigations. Causal analysis is challenging because aging can not be reversed and many factors are mixed after surgery, such as gut hormones, microbiota, bile acids and yet-to-be identified signal molecules. Besides, the phenomenon and mechanisms might differ vastly between species. Despite further studies about aging is needed, our study verified the compromising improvement of glucose metabolism in aged rats, and aging might moderate the effect of RYGB. An investigation about chaperone proteins is being planned, and we hope it can reveal the effect of aging on RYGB bariatric surgery.

## Conclusion

RYGB could induce a glucose metabolism improvement in aged diabetic rats, and aging might moderate the effect of RYGB.

## Data Availability

Authors can confirm that all relevant data are included in the article and/or its supplementary information files.
